# Pleiotropic Effects of DDT Resistance on Male Size and Behaviour

**DOI:** 10.1007/s10519-017-9850-6

**Published:** 2017-05-02

**Authors:** Wayne G. Rostant, Jemima Bowyer, Jack Coupland, James Facey, David J. Hosken, Nina Wedell

**Affiliations:** 1 Biosciences, University of Exeter, Penryn Campus, Penryn, TR10 9FE Cornwall UK; 2 College of Engineering, Mathematics and Physical Sciences, University of Exeter, Streatham Campus, Exeter, EX4 4QF Devon UK; 3 School of Biological Sciences, University of East Anglia, Norwich, NR4 7TJ Norfolk UK

**Keywords:** Mating success, Insecticide resistance, Aggression, Courtship, Body size, Pleiotropy

## Abstract

Understanding the evolution and spread of insecticide resistance requires knowing the relative fitness of resistant organisms. In the absence of insecticides, resistance is predicted to be costly. The *Drosophila melanogaster* DDT resistance allele (DDT-R) is associated with a male mating cost. This could be because resistant males are generally smaller, but DDT-R may also alter courtship behaviours. Here we tested for body size and courtship effects of DDT-R on mating success in competitive and non-competitive mating trials respectively. We also assessed relative aggression in resistant and susceptible males because aggression can also influence mating success. While the effect of DDT-R on male size partly contributed to reduced mating success, resistant males also had lower rates of courtship and were less aggressive than susceptible males. These differences contribute to the observed DDT-R mating costs. Additionally, these pleiotropic effects of DDT-R are consistent with the history and spread of resistance alleles in nature.

**Electronic supplementary material:**

The online version of this article (doi:10.1007/s10519-017-9850-6) contains supplementary material, which is available to authorized users.

## Introduction

A key question in the evolution and spread of insecticide resistance is the fitness of organisms carrying a resistance allele. Theory holds that, in the absence of insecticide, resistance should be costly (Crow 1957). However, evidence of pleiotropic fitness costs associated with insecticide resistance alleles is equivocal. Some studies have found that investment in resistance carries a fitness cost (Minkoff and Wilson 1992; Chevillon et al. 1997; Boivin et al. 2001; Berticat et al. 2002; Rivero et al. 2011; Smith et al. 2011; Platt et al. 2015), whereas others have failed to find any detrimental effects (Follett et al. 1993; Tang et al. 1999; Castañeda et al. 2011), and some have even demonstrated insecticide resistance alleles conferring pleiotropic fitness benefits (Omer et al. 1992; Arnaud and Haubruge 2002; McCart et al. 2005; Bielzaet al. 2008). Furthermore, pleiotropic effects of resistance can be positive or negative, depending on the precise fitness components measured (Brewer and Trumble 1991), and these effects can also be sex-specific (Smith et al. 2011). Finally, resistance alleles can also show epistasis, where pleiotropic effects are mediated by the genotype (genetic background) of the insect (Hollingsworth et al. 1997; Oppert et al. 2000; Smith et al. 2011).

Both epistasis and sex-specific fitness effects have recently been reported for a DDT resistance allele in *Drosophila melanogaster* (McCart et al. 2005; Smith et al. 2011; Rostant et al. 2015; also see; Hawkes et al. 2016). DDT resistance in *D. melanogaster* is conferred by the upregulation of a cytochrome P450 enzyme, CYP6G1 (Daborn et al. 2002). Resistant flies have tandemly duplicated C*yp6g1* alleles that possess the Long Terminal Repeat (LTR) of an *Accord* retrotransposon inserted in the cis-regulatory region (Daborn et al. 2002). While there appears to be a benefit to females of carrying this resistant allele (DDT-R) (McCart et al. 2005), a recent study (Smith et al. 2011) demonstrated a strong competitive mating disadvantage for DDT-R males in the *Canton-S* (CS) background (for additional evidence also see Rostant et al. 2015 and; Hawkes et al. 2016). This may be because resistant males are smaller than susceptible males (Smith et al. 2011): body size is positively associated with male fitness in *D. melanogaster* (Partridge and Farquhar 1983; Partridge et al. 1987; Pitnick 1991). However, this does not preclude the possibility that DDT-R could also affect other components of mating success, especially because resistance alleles affect behaviour (Rowland 1991; Foster et al. 2007, 2011).

Here, we test the size-mediated effect of DDT-R on competitive mating success and examine DDT-R effects on aspects of male behaviour. We initially conducted competitive mating trials, directly manipulating the size disparity between resistant and susceptible males, to investigate whether the size difference is sufficient to cause the DDT-R mating disadvantage. Secondly, we examined the courtship behaviour of DDT-R and susceptible males in a non-competitive context to quantify potential differences in the intensity, rate and sequence of behaviours that could generate differential mating success. Lastly, we investigated male–male aggression to see if DDT-R males differed from susceptible males (Dierick and Greenspan 2006).

## Materials and methods

### Introgression and population maintenance

CS stock flies were initially homozygous for the ancestral (susceptible) *Cyp6g1* allele. The DDT-R allele *Cyp6g1-BA* (Schmidt et al. 2010) was introgressed using a separate wild-caught resistant strain for the initial cross (Smith et al. 2011). This was followed by repeated backcrossing for seven additional generations into stock CS flies. After each generation of backcrossed mating, developing progeny were subject to DDT selection by lacing rearing vials with 500 µL of 4 μg/mL DDT in acetone solution. Effectively, the dose is 2 µg of DDT per vial, which has been shown to result in close to 90% 24-h mortality in CS flies (Daborn et al. 2001). After the backcrossing, mating pairs were established and the progeny of homozygous resistant crosses (RR × RR: PCR diagnostic according to Daborn et al. (2002)) were subsequently used to found the corresponding DDT-R population (CS_RR_). Both populations (CS_RR_ and susceptible, CS_SS_) were subsequently maintained at 25 °C on complete Jazz-mix *Drosophila* food (Fisher, Pittsburgh, PA, USA) in 30 × 30 × 30 cm population cages with 12:12 h light:dark and humidity ~40%.

Experimental flies were collected as first instar larvae from Petri dishes containing 1.5% agar in apple juice with yeast paste spread on a small area of the surface. With the exception of the size manipulation experiment, larvae were reared at a standard density of 100 larvae per food vial (approximately 5 mL in 3 × 7 cm vials). Virgin adult flies were held in narrow food vials (approximately 5 mL in 2 × 9.5 cm circular vials) at a density of approximately 20 flies per vial.

### Effect of size and resistance allele on mating success

To obtain males of various sizes for this experiment, larvae of both genotypes were reared at two different densities of either 25 per vial or 150 flies per vial. Twenty-four hours before the experiment, we anaesthetised (using CO_2_) 2–4-day old virgin CS_RR_ and CS_SS_ males and sorted them, under a dissecting microscope, into categories according to thorax length measurements. Preliminary measurements had given modal thorax lengths of 1.07 mm for susceptible males and 0.98 for resistant males. We used these to define the three broad size categories (‘large’≥1.07; 1.07>’medium’>0.98 mm; ‘small’≤0.98 mm). Individual large males of each genotype were then randomly paired with small males of the other, as were medium resistant with medium susceptible.

Each pair was gently aspirated into a narrow polypropylene vial. Prior to this pairing off, we used blue and pink paint powder to identify individual males in a factorial way (Champion de Crespigny and Wedell 2007; Smith et al. 2011) so that half the resistant and susceptible males were blue and the other half were pink. Thus pink males always competed against blue males, and resistant males always competed against susceptible males. Experimental observers were blind to these treatments. On the day of the mating assay a single virgin female was gently aspirated into each vial. Females were 3–5 days old and of a wild-type background (Dahomey) into which the recessive *sparkling poliert* (*spa*) mutation had been recently backcrossed (Fricke et al. 2009). This tester strain was used for consistency with previous studies on the effect of DDT-R on male competitive fitness (Smith et al. 2011). A number of different mating assays were conducted in Smith et al. (2011), some of which involved sperm competition (and thus required scoring of offspring to determine paternity). Rather than use different tester females for the different tests, we opted for consistency within the previous study and with this, our follow-up. For each replicate triad, at the onset of copulation we immediately aspirated the unsuccessful male out of the vial and similarly removed the successful male post-copulation. Wing size was measured as a surrogate of body size for all successful and unsuccessful males using SPOT BASIC 4.1 (Diagnostic instruments, Inc., Sterling Heights, MI, USA).

### Male courtship behaviour

Replicates of four homozygous crosses (CS_RR_ ♀ × CS_RR_ ♂, CS_RR_ ♀ × CS_SS_ ♂, CS_SS_ ♀ × CS _SS_ ♂, CS_SS_ ♀ × CS_RR_ ♂) were established. Each dyad consisted of one virgin male and one virgin female in a shallow cylindrical arena, with courtship being video recorded from above. Each arena consisted of a small plastic Petri dish 3.5 × 1 cm (diameter × depth) with a secure lid and containing a small food cup (1.5 mL Eppendorf cap) (Dierick and Greenspan 2006). The food cup was filled with 2.0% agar in apple juice with yeast paste spread on a small area of the surface. Eight of these arenas could be arranged, in a 2 × 4 array, within the maximum field of view which allowed detailed recording of courtship behaviour under ambient light. Arenas were separated from each other by white paper partitions. Twelve hours prior to each assay virgin females were aspirated into each arena to adjust to their surroundings and immediately prior to loading the males the array was placed under a high definition video camera (Panasonic HD-SD90). Recording commenced and males were then aspirated into each arena. Once a pair began copulating the arena was removed and replaced in the array by a new arena containing another virgin female, repeating the assay. If there was no copulation after 30 min the arena was removed and the male was classed as unsuccessful. Successful males were retained for size measurement as above. All flies were 6 days old at the time of assay.

Behavioural recordings were analysed for 13 successful pairings of each cross. Seven courtship behaviours were distinguished following the protocol of Ejima and Griffith (2007) (Supplementary table S1). Continuous records were analysed, and the frequency and duration of each behaviour, as well as the times at which each behaviour stopped and started, was recorded.

### Male aggression

Within-genotype aggression was video recorded between pairs of virgin CS_SS_ and CS_RR_ males within the arena setup described above, with the exception that a decapitated female was placed on the food surface of each arena immediately prior to the assay to aid in attracting males (Chen et al. 2002). The resistance status of the decapitated females in each arena was balanced across male genotypes. Flies reared in social environments have suppressed aggression (Hoffmann 1990), but this is reversible after just 1 day of isolation (Wang et al. 2008). Therefore experimental flies were individually isolated 24 h before each assay. To further increase aggression levels, each individual male was then transferred, 90 min before each assay, into foodless vials containing water-saturated cotton wool. This time-scale has been shown to increase aggression without revealing any underlying differences in starvation sensitivity (Edwards et al. 2006).

All flies were 5–8 days old during the experiment and were not exposed to anaesthesia for at least 24 h prior to the assay. As in the courtship behaviour assay, an array of eight arenas (maximum) at a time was recorded. Two males of the same genotype (CS_RR_ or CS_SS_) were gently aspirated into each arena. The flies were allowed to adjust for 15 min, and were then recorded for 10 min using the same camera as in the courtship behaviour assay. Flies were then anesthetised and retained for size measurement as per the male size-effect assay. In this manner a total of 30 replicate pairs of each genotype were assayed for aggression. Four separate aggressive behaviours were defined following Chen et al. (2002) (Supplementary table S1). From each 10 min recording, the number of aggressive behavioural occurrences was noted.

### Statistical analyses

Statistical analyses were performed in R 3.2.3 (R Core Team (2015) using the base stats package, except where otherwise stated. For univariate behavioural count and duration data we used generalized linear models (GLMs); or Generalized linear mixed-effects models (GLMMs) as implemented in package ‘lme4’ (Bates et al. 2015). Maximal models included male- and, where appropriate female-, resistance genotype as explanatory variables with male size as a covariate. Wherever appropriate, non-normal error structure was specified with default link functions. Overdispersion was accounted for by using quasi-likelihood to specify more appropriate variance functions. In all GLM or GLMM analyses stepwise model simplification of the maximal model with analysis of deviance was used to determine significant terms. Significance was adjusted for multiple univariate testing of courtship behaviours using the Benjamini-Hochberg method to control for false discovery rate (Benjamini and Hochberg 1995).

Overall courtship behavioural response was analysed within a compositional framework by permutational multivariate analysis of variance, using the adonis2() function in the ‘vegan’ package (Oksanen et al. 2017). Prior to analysis, time spent in each courtship behaviour by each courting pair (sample) was transformed via the Chi square distance transformation in function decostand(), and a pairwise dissimilarity matrix constructed based on Euclidean distances. Use of Chi square distances has been shown to have favourable properties in the analysis of compositions (Jackson 1997), particularly when there are many essential zeros (Stewart 2016) as is the case with our behavioural data. After checking for multivariate homogeneity of group variances using function betadisper(), the dissimilarity matrix was then subjected to permutational MANCOVA with all the same explanatory terms as in the univariate GLMs. Significance of terms was determined by stepwise model simplification of the maximal model using marginal permutation tests, with pseudo-*F* ratios (McArdle and Anderson 2001).

Courtship behavioural sequences were analysed as discrete event single-order Markov Chains, testing for the existence of non-random temporal associations among the seven different behaviours. Transition matrices were constructed by tabulating all instances in which one behaviour led to another. These were pooled for all males of each genotype to give two overall transition matrices, one for resistant males and one for susceptible males. Transition categories that never occurred (e.g. decamp→lick) were considered structural zeros (West and Hankin 2008) and not included in subsequent analysis. A generalisation of Fisher’s Exact test which can cope with structural zeros is implemented in R package ‘aylmer’ (West and Hankin 2008) and was used to test for non-randomness (stereotypical structure) in the sequence of behaviours both at the level of the whole matrix and for each possible transition. Markov Chain Monte Carlo (MCMC) was used to explore the space of permissible matrices and approximate the *p* value (West and Hankin 2008).

## Results

### Effects of size and resistance allele on mating success

**Fig. 1:**
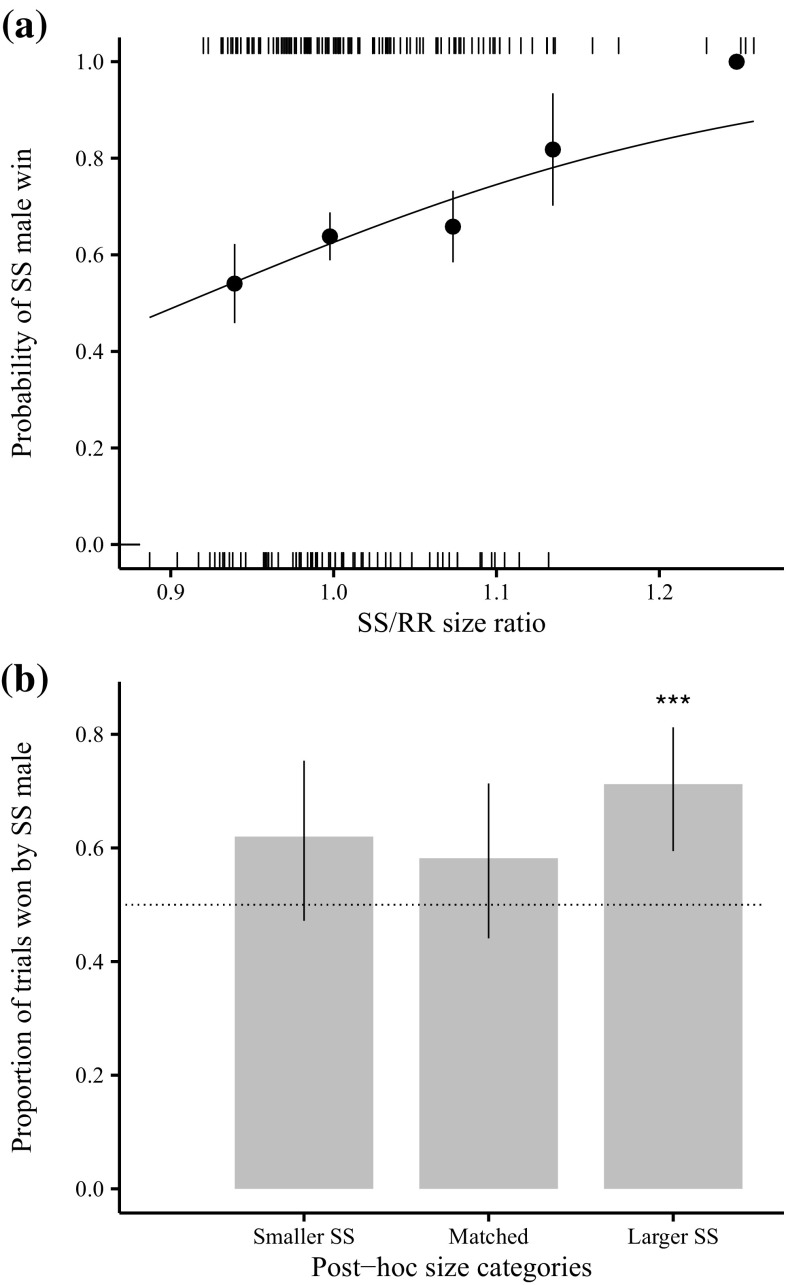
The effect of relative size on whether a susceptible or resistant male wins in competitive trials. **a** Logistic plot: *the curve* represents the fit of the logistic model of susceptible male win probability as a function of the susceptible/resistant wing size ratio (SS/RR). Points show empirical probabilities (+/− s.e.) of a susceptible male win. Rugs at the* top and bottom* of the graph show the empirical distribution of binary win data. (**b**) Probability of susceptible male win, with 95% binomial confidence intervals, when competitive trial data is divided into three post-hoc categories. Asterisks represent significant departure from expectation of 50% (Exact binomial test) *indicated by dotted line*: *** *p* < 0.001

Of the 187 successful competitive trials, susceptible males won the majority (120) of matings. A maximal GLM model of the binary response (susceptible or resistant male wins) was fitted as a function of size ratio (i.e. susceptible male wing size/resistant male wing size), along with susceptible male wing size as a covariate and susceptible male colour with interactions, using binomial error structure. Stepwise model simplification revealed a sole significant main effect of the size ratio on whether a resistant or susceptible male won a competitive trial (Fig. 1a; χ^2^_1_= 5.204, *p* = 0.023, binomial errors). Susceptible males have a greater than 50% chance of winning a competitive trial when the susceptible/resistant size ratio is at least 0.9. Further examination was carried out by dividing the trials by post-hoc wing size measurements into three categories: “Matched”, which consisted of closely sized males (within ± 2.5% of each other); “Smaller SS”, where the susceptible male was more than 2.5% smaller than the resistant; and “Larger SS”, where the susceptible was more than 2.5% larger than the resistant. In the latter category susceptible males won the significant majority of trials (Exact Binomial Test, 52 successes from 73 trials, *p* < 0.001) but there was no significant departure from a null of 50% for either the “Matched” (Exact Binomial Test, 32 successes from 55 trials, *p* = 0.28) or “Smaller SS” (Exact Binomial Test, 31 successes from 50 trials, *p* = 0.12) categories (Fig. 1b). Thus there is nullification, but no reversal of the susceptible mating advantage when resistant males are larger than susceptible males.

Model simplification of log-transformed copulation latency as a function of wing size ratio and susceptible male colour yielded a null minimum adequate model. Thus the size difference of the competing males did not have any effect on copulation latency (log-transformed latency, *F*_1,185_ = 1.751, *p* = 0.19, normal errors).

### Male courtship behaviour

Both resistant and susceptible males displayed the full repertoire of courtship behaviours (Ejima and Griffith 2007). However, two behaviours were very rare (fencing: 81% zero cases; tapping: 73% zero cases) and so were removed from subsequent multivariate and univariate analyses. Prior to permutational MANCOVA on transformed behavioural data, multivariate outliers were detected and the worst six removed to minimize their influence on subsequent tests. These samples coincided with courtship durations <45 s long and were equally distributed between RR and SS male treatments. Their removal ensured multivariate homogeneity of variances, which was confirmed for groups defined both by male resistance status (Permutation dispersion test, pseudo-*F*_1,44_ = 1.414, N. perm = 999, *p* = 0.243) and female resistance status (Permutation dispersion test, pseudo-*F*_1,44_ = 0.091, N.perm = 999, *p* = 0.788). After stepwise removal of all other explanatory terms due to non-significance, there was a significant multivariate effect of male resistance status (Permutational MANOVA marginal test, pseudo-*F*_1,43_ = 4.550, N.perm = 2 × 10^5^, *p* = 0.012) and a marginally significant effect of female resistance (Permutation MANOVA marginal test, pseudo- *F*_1,43_ = 3.006, N.perm = 2 × 10^5^, *p* = 0.048) on courtship behaviour.

**Fig. 2:**
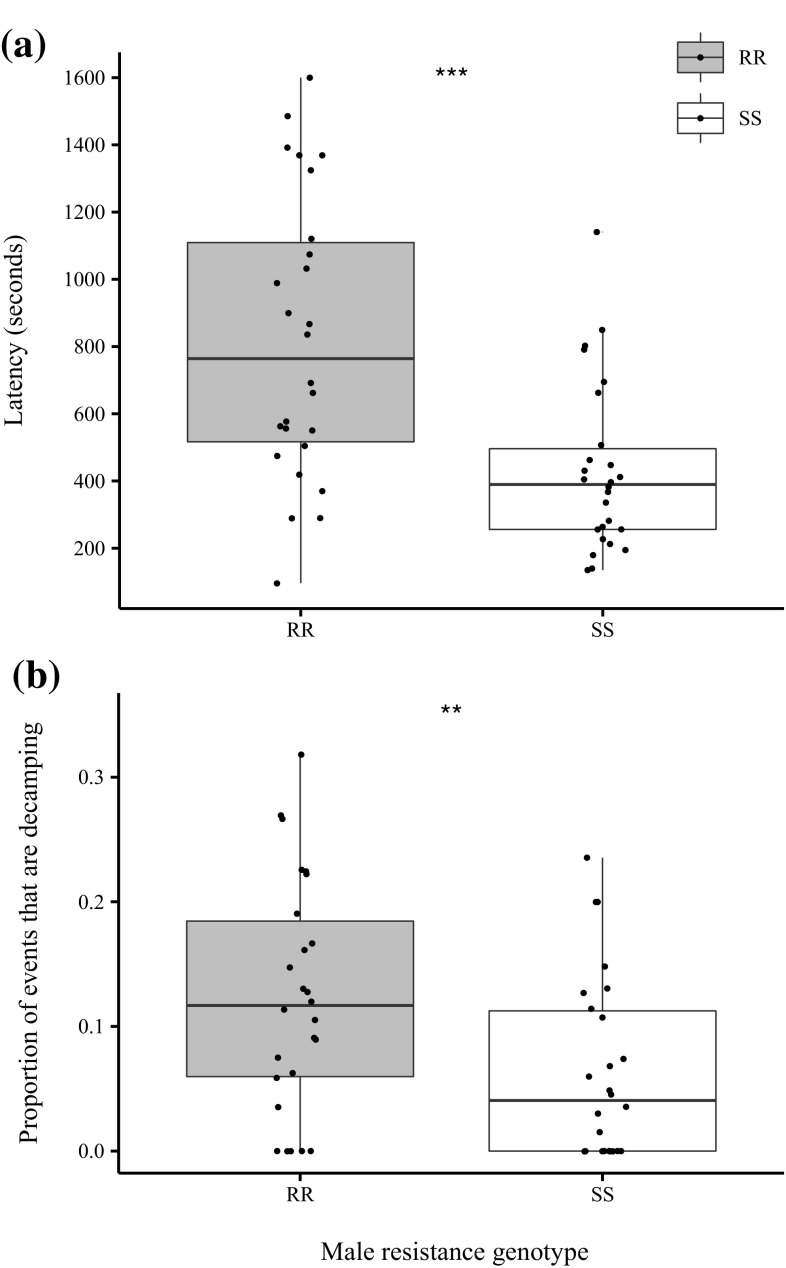
Effect of male resistance genotype on **a** total copulation latency, and **b** the proportion of behavioural events that are decamping events. *Asterisks* represent significance of main effect of male genotype in GLM: ** *p* < 0.01; *** p < 0.001

**Fig. 3:**
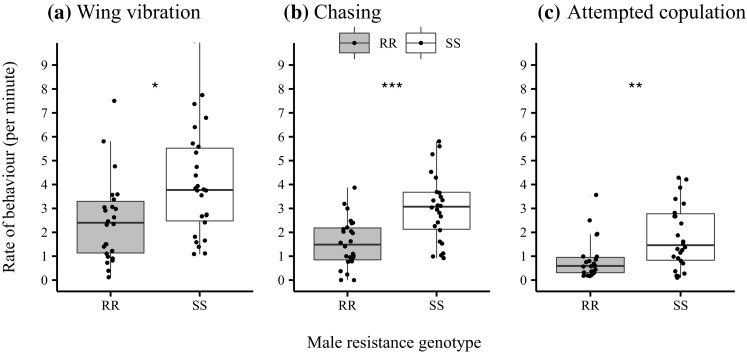
Effect of male resistance genotype on rates (min^−1^) of three common courtship behaviours **a** wing vibration, **b** chase, and **c** attempted copulation. *Asterisks* represent significance of main effect of male genotype in GLM: * *p* < 0.05; ** *p* < 0.01; *** *p* < 0.001

None of the GLM models revealed any significant effects of female resistance status and male size, nor were any interactions that included these terms. However, male resistance status altered copulation latency and this effect was driven by time from first courtship to copulation i.e. ‘courtship duration’ (Table 1). Thus resistant males are slower to copulate once courtship has commenced (Fig. 2a). Resistant males also decamped more (Fig. 2b), had lower rates of wing vibration (Fig. 3a), chasing (Fig. 3b) and copulation attempts (Fig. 3c).

**Table 1:** Summary of courtship behavioural responses to possession of DDT-R allele. ↑ represents increase in resistant males relative to susceptible males

Behavioural response	Measure	Effect (RR male relative to SS)	Test summary Test, Error family, test statistic *p* value, (adjusted *p* value)
Copulation latency	Absolute (seconds)	↑	GLM, gamma, *F*_1,50_ = 14.236 *p* < 0.001, (*p*_*adj*_ = 0.004)
Courtship latency	Absolute (seconds)	–	GLM, quasipoisson, *F*_1,50_ = 0.8472 *p* = 0.36, (*p*_*adj*_ = 0.473)
Courtship duration	Absolute (seconds)	↑	GLM, quasipoisson, *F*_1,50_ = 11.471 *p* = 0.001, (*p*_*adj*_ = 0.008)
Decamping	Proportion of time	–	GLM, quasibinomial, *F*_1,50_ = 2.3412 *p* = 0.132, (*p*_*adj*_ = 0.225)
	Relative frequency	↑	GLM, quasibinomial, *F*_1,50_ = 7.959 *p* = 0.007, (*p*_*adj*_ = 0.023)
Wing vibration	Proportion of time (logit-transformed)	–	GLM, Gaussian, *F*_1,50_ = 3.1183 *p* = 0.082, (*p*_*adj*_ = 0.175)
	Relative frequency	–	GLM, binomial, χ ^2^_1_= 0.47196 *p* = 0.49, (*p*_*adj*_ = 0.598)
	Rate (min^−1^)	↓	GLM, gamma, *F*_1,49_ = 6.831 *p* = 0.012, (*p*_*adj*_ = 0.034)
Chasing	Proportion of time	–	GLM, quasibinomial, *F*_1,50_ = 0.0671 *p* = 0.797, (*p*_*adj*_ = 0.903)
	Relative frequency (logit-transformed)	–	GLM, Gaussian, *F*_1,50_ = 1.012 *p* = 0.319, (*p*_*adj*_ = 0.452)
	Rate (min^−1^)	↓	GLM, Gaussian, *F*_1,49_ = 17.934 *p* < 0.001, (*p*_*adj*_ = 0.004)
Attempted copulation	Absolute (count)	–	GLM, quasipoisson, *F*_1,50_ = 0.003 *p* = 0.96, (*p*_*adj*_ = 0.990)
	Relative frequency (logit-transformed)	–	GLM, Gaussian, *F*_1,50_ = 1.470 *p* = 0.230, (*p*_*adj*_ = 0.355)
	Rate (min^−1^)	↓	GLM, gamma, *F*_1,48_ = 9.049 *p* = 0.004, (*p*_*adj*_ = 0.019)
Genital licking	Proportion of time	–	GLM, quasibinomial, *F*_1,50_ = 4.369 *p* = 0.042, (*p*_*adj*_ = 0.102)
	Relative frequency	–	GLM, binomial, χ^2^_1_= 0.0002 *p* = 0.986, (*p*_*adj*_ = 0.990)
	Rate (min^−1^)	–	Wilcoxon rank-sum test, *W* = 252, *Z* = -1.580 *p* = 0.12, (*p*_*adj*_ = 0.225)

↓ Represents decrease in resistant males relative to susceptible males

Dash indicates no difference between resistant and susceptible males

GLM error family (with any transformations of response variable), test statistic and *p* values given, except in the case of genital licking rate for which a nonparametric test was required

Adjusted *p* values (*p*_*adj*_) are Benjamini–Hochberg corrected for multiple testing

Twenty-nine different behavioural transitions were observed, the most frequent being chase→ wing vibration (resistant count = 246; susceptible count = 192) and wing vibration→attempt copulation (resistant count = 79; susceptible count = 81). Results of the generalised Fisher’s Exact Test show departure from independence for both the resistant (*p* < 0.001) and susceptible (*p* < 0.001) matrices, indicating the presence of stereotypical behavioural sequences. All significant transitions are shown in kinematic diagrams of resistant and susceptible male courtship behaviour (Supplementary Fig. S1). Overall patterns of behaviour were similar for both genotypes with males tending to move from chasing to wing vibration followed by genital licking and/or attempted copulation. When an attempt failed, the male would chase the female if she moved away, or transition back to wing vibration. Key differences in the patterns of the two male genotypes include transitions away from and returning to the female (i.e. decamping). Resistant males were more likely to decamp following a chase with a significant 19% of resistant chases ending with the male decamped (Supplementary Table S2) as opposed to a non-significant 7% of susceptible chases (Supplementary Table S3).

### Aggression

**Fig. 4:**
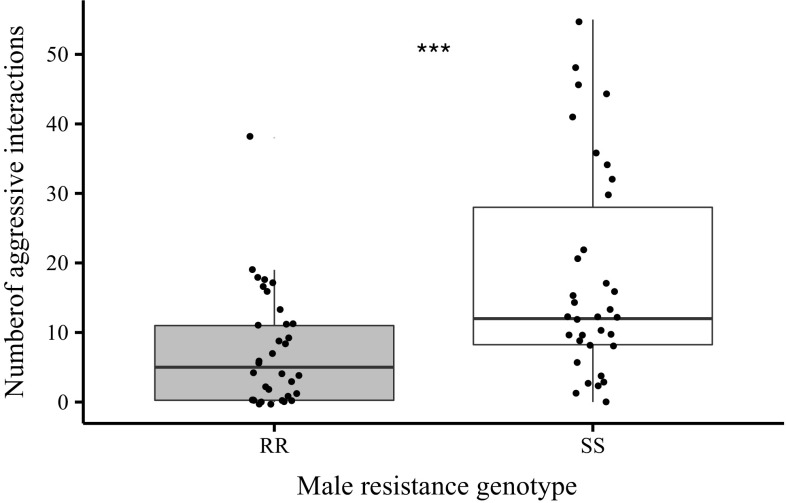
Counts of all aggressive behaviours observed in pairs of resistant and susceptible males. *Asterisk* represents significance of main effect of male genotype in GLMM: *** *p* < 0.001

Thirty-four pairs of each male genotype were assayed for aggression. Aggressive behaviours were observed in 33 of the susceptible pairs and 25 of the resistant pairs, revealing a significant association between male genotype and the presence of aggression (Fisher’s Exact test, *p* = 0.013). Complete wing size data was obtained for 60 of the 68 pairs, permitting the size disparity between males to be calculated. A maximal GLMM model of the total number of aggressive behaviours was fitted as a function of male genotype, decapitated female genotype and size disparity with all interactions, using a negative binomial error structure and time of day as a random factor with three levels (morning, afternoon, evening). The minimal adequate model included only male genotype as a significant factor (Fig. 4; χ^2^_1_ = 15.512, *p* < 0.001, negative binomial errors). While resistant males displayed lower aggression than susceptible males, disparity in size between competing males had no effect on total aggression levels. Similarly there was no effect of size disparity, male genotype or their interactions on the proportion of aggressive acts that were high intensity (boxing and head butting) as opposed to low intensity (wing threat and chase).

## Discussion

DDT-R can have sexually antagonistic fitness effects in the absence of DDT (Smith et al. 2011; Rostant et al. 2015; Hawkes et al. 2016), but the phenotypic cause of lower fitness in DDT-R males is not clear. Here we show that the effect of DDT-R on male size previously documented (Smith et al. 2011) is an important mediator of the mating cost for DDT-R males, but is insufficient to explain the magnitude of this cost found in the *Canton-S* genetic background. We also identified differences in courtship and aggression between resistant and susceptible males that are likely to also contribute to differential male mating success. Our previous results (Smith et al. 2007; Rostant et al. 2015) suggested that the DDT-R mating disadvantage was a possible outcome of the DDT-R size effect. Here, by directly manipulating the relative sizes of competing males, we confirmed that male size influences the probability of winning competitive mating trials. Moreover, we show that reversal of the DDT-R size disparity eliminates the mating disadvantage of these males. However, if the competitive mating disadvantage conferred to DDT-R males was solely a result of pleiotropic size effects of carrying the resistance allele, then larger resistant males should have a competitive advantage against smaller susceptible males. This was not seen. In fact, large resistant males still lost 62% of their trials against small susceptible males, although the probability of resistant males winning a trial does not exceed 50% until the susceptible/resistant size ratio drops below 0.9. This suggests an effect of DDT resistance status on male competitive mating success over and above the effect of DDT-R on size.

Our analysis of courtship suggests why this might be, because resistant males showed a two-fold increase in copulation latency compared to susceptible males. Copulation latency is one measure of male-attractiveness (Taylor et al. 2008; Okada et al. 2011) indicating that DDT-R males are less attractive. This points towards differences in other key behaviours in the lead up to successful intromission (Table 1) with resistant males performing courtship song (wing vibration) at a lower rate and chasing females at a lower rate. In fact, male resistance status had an overall significant multivariate effect on courtship behaviour. There is also the possibility that DDT-R also alters fly cuticular hydrocarbons, another trait that affects male attractiveness (Ingleby et al. 2014). Interestingly, while we also detected a marginally significant multivariate effect of female resistance on courtship behaviour, subsequent univariate tests failed to indicate any effect on specific behaviours, suggesting more subtle differences that may require a fine-grained examination of interactions from the female perspective and/or greater replication.

Decamping (effectively aborting mating attempts already initiated) was the major behavioural difference between resistant and susceptible males. This suggests differences in the structure of courtship caused by DDT-R and this is borne out in the behavioural sequence analysis. Overall transition matrices were found to be significantly non-random, consistent with well documented stereotypical sequences of courtship behaviour (Spieth 1974). However, while the overall sequences of behaviour were similar for both male genotypes, there was a much higher probability of a DDT-R male’s chase ending in decamping and these males decamp more often than by chance and much more often than susceptible males. Furthermore, susceptible males were more likely to follow courtship song (as indicated by wing movement) with a copulation attempt than the DDT-R males. This disrupted courtship sequence and higher incidence of decamping probably accounts for the increased copulation latency and lower mating success of DDT-R males.

Aggression levels were also much lower in DDT-R males. While these results were stark, it is worth noting that the experimental protocol maximised aggression levels by priming males before the trial (through isolation and starvation). It is possible therefore that differences in realised aggression may not be as apparent in other social or environmental contexts. Nonetheless this finding could also explain fitness decreases in DDT-R males as previous observations suggest that aggression can confer a mating advantage for territorial males (Hoffmann and Cacoyianni 1990; Baxter et al. 2015).

To date the underlying developmental and genetic pathways by which DDT-R affects male size, aggression and courtship behaviour are not clear. However it seems apparent that upregulation of *Cyp6g1* influences both male size and behaviour in the CS background. This inference is corroborated by findings in another genetic background (Ives) where male genotypes with low competitive mating success had significantly higher expression of *Cyp6g1* irrespective of DDT-R (which was not examined) (Drnevich et al. 2004). Future transcriptome studies that include quantifying the expression levels of CYP6G1 and other genes implicated in regulating behaviours in resistant and susceptible CS flies are needed to evaluate their association with male reproductive behaviours and size variation (and see Hawkes et al. 2016).

The present study suggests that both male–male competition and female choice influence the mating success of DDT-R males. As yet it is not clear how the different aspects of DDT-R-male phenotype are integrated to cause the observed pre-copulatory mating cost. However, we have provided evidence of multiple effects of DDT-R on male behaviours closely linked to fitness and confirm the mating cost previously reported for DDT-R males is at least partly mediated by pleiotropic size and behavioural effects. These differences are likely to explain why DDT-R did not fix prior to the use of DDT despite increasing female fitness (Rostant et al. 2015).

## Electronic supplementary material

Below is the link to the electronic supplementary material.

Fig. S1 Kinematic diagram of behavioural transitions that occurred more than 10% of the time for (a) susceptible males and (b) resistant males during courtship. Arrow thickness indicates probability of occurrence. Solid, black arrows represent those transitions which occurred more frequently than expected by chance (*p* < 0.05) and grey dashed arrows show non-significant transitions (*p* < 0.05). Box size indicates frequency of behaviour. (PDF 182 KB)

Supplementary material 2 (DOCX 12 KB)

Supplementary material 3 (DOCX 12 KB)

Supplementary material 4 (DOCX 12 KB)

## References

[CR1] Arnaud L, Haubruge E (2002). Insecticide resistance enhances male reproductive success in a beetle. Evol Int J org Evol.

[CR2] Bates D, Maechler M, Bolker B, Walker S (2015). Fitting linear mixed-effects models using lme4. J Stat Softw.

[CR3] Baxter CM, Barnett R, Dukas R (2015). Aggression, mate guarding and fitness in male fruit flies. Anim Behav.

[CR4] Benjamini Y, Hochberg Y (1995). Controlling the false discovery rate: a practical and powerful approach to multiple testing. J R Stat Soc B.

[CR5] Berticat C, Boquien G, Raymond M, Chevillon C (2002). Insecticide resistance genes induce a mating competition cost in *Culex pipiens* mosquitoes. Genet Res.

[CR6] Bielza P, Quinto V, Grávalos C, Abellán J, Fernández E (2008). Lack of fitness costs of insecticide resistance in the western flower thrips (Thysanoptera: Thripidae). J Econ Entomol.

[CR7] Boivin T, Chabert d’Hières C, Bouvier JC, Beslay D, Sauphanor B (2001). Pleiotropy of insecticide resistance in the codling moth, *Cydia pomonella*. Entomol Exp Appl.

[CR8] Brewer MJ, Trumble JT (1991). Inheritance and fitness consequences of resistance to fenvalerate in *Spodoptera exigua* (Lepidoptera, Noctuidae). J Econ Entomol.

[CR9] Castañeda LE, Barrientos K, Cortes PA, Figueroa CC, Fuentes-Contreras E, Luna-Rudloff M, Silva AX, Bacigalupe LD (2011). Evaluating reproductive fitness and metabolic costs for insecticide resistance in *Myzus persicae* from Chile. Physiol Entomol.

[CR10] Champion de Crespigny FEC, Wedell N (2007). Mate preferences in *Drosophila* infected with *Wolbachia*?. Behav Ecol Sociobiol.

[CR11] Chen S, Lee AY, Bowens NM, Huber R, Kravitz EA (2002). Fighting fruit flies: a model system for the study of aggression. Proc Natl Acad Sci USA.

[CR12] Chevillon C, Bourguet D, Rousset F, Pasteur N, Raymond M (1997). Pleiotropy of adaptive changes in populations: Comparisons among insecticide resistance genes in *Culex pipiens*. Genet Res.

[CR13] Crow JF (1957). Genetics of insect resistance to chemicals. Annu Rev Entomol.

[CR14] Daborn P, Boundy S, Yen J, Pittendrigh B, ffrench-Constant R (2001). DDT resistance in *Drosophila* correlates with Cyp6g1 over-expression and confers cross-resistance to the neonicotinoid imidacloprid. Mol Genet Genom.

[CR15] Daborn PJ, Yen J, Bogwitz MR, Le Goff G, Feil ES, Jeffers S, Tijet N, Perry T, Heckel D, Batterham P, Feyereisen R, Wilson TG (2002). A single P450 allele associated with insecticide resistance in *Drosophila*. Science.

[CR16] Dierick HA, Greenspan RJ (2006). Molecular analysis of flies selected for aggressive behavior. Nat Genet.

[CR17] Drnevich JM, Reedy MM, Ruedi EA, Rodriguez-Zas S, Hughes KA (2004). Quantitative evolutionary genomics: differential gene expression and male reproductive success in *Drosophila melanogaster*. Proc R Soc Lond B Biol Sci.

[CR18] Edwards AC, Rollmann SM, Morgan TJ, Mackay TFC (2006). Quantitative genomics of aggressive behavior in *Drosophila melanogaster*. PLoS Genet.

[CR19] Ejima A, Griffith LC (2007) Measurement of courtship behavior in *Drosophila melanogaster*. CSH Protocols. doi:10.1101/pdb.prot4847 10.1101/pdb.prot4847 21356948

[CR20] Follett PA, Gould F, Kennedy GG (1993). Comparative fitness of three strains of Colorado potato beetle (Coleoptera, Chrysomelidae) in the field—Spatial and temporal variation in insecticide selection. J Econ Entomol.

[CR21] Foster SP, Tomiczek M, Thompson R, Denholm I, Poppy G, Kraaijeveld AR, Powell W (2007). Behavioural side-effects of insecticide resistance in aphids increase their vulnerability to parasitoid attack. Anim Behav.

[CR22] Foster SP, Denholm I, Poppy GM, Thompson R, Powell W (2011). Fitness trade-off in peach-potato aphids (*Myzus persicae*) between insecticide resistance and vulnerability to parasitoid attack at several spatial scales. Bull Entomol Res.

[CR23] Fricke C, Wigby S, Hobbs R, Chapman T (2009). The benefits of male ejaculate sex peptide transfer in *Drosophila melanogaster*. J Evol Biol.

[CR24] Hawkes MF, Gamble CE, Turner ECR, Carey MR, Wedell N, Hosken DJ (2016). Intralocus sexual conflict and insecticide resistance. Proc R Soc B.

[CR25] Hoffmann AA (1990). The influence of age and experience with conspecifics on territorial behavior in *Drosophila melanogaster*. J Insect Behav.

[CR26] Hoffmann AA, Cacoyianni Z (1990). Territoriality in *Drosophila melanogaster* as a conditional strategy. Anim Behav.

[CR27] Hollingsworth RG, Tabashnik BE, Johnson MW, Messing RH, Ullman DE (1997). Relationship between susceptibility to insecticides and fecundity across populations of cotton aphid (Homoptera: Aphididae). J Econ Entomol.

[CR28] Ingleby FC, Hosken DJ, Flowers K, Hawkes MF, Lane SM, Rapkin J, House CM, Sharma MD, Hunt J (2014). Environmental heterogeneity, multivariate sexual selection and genetic constraints on cuticular hydrocarbons in *Drosophila simulans*. J Evol Biol.

[CR29] Jackson DA (1997). Compositional data in community ecology: the paradigm or peril of proportions?. Ecology.

[CR30] McArdle BH, Anderson MJ (2001). Fitting multivariate models to community data: a comment on distance-based redundancy analysis. Ecology.

[CR31] McCart C, Buckling A, ffrench-Constant RH (2005). DDT resistance in flies carries no cost. Curr Biol.

[CR32] Minkoff C, Wilson TG (1992). The competitive ability and fitness components of the *Methoprene-tolerant (Met) Drosophila* mutant resistant to juvenile hormone analog insecticides. Genetics.

[CR33] Okada K, Blount JD, Sharma MD, Snook RR, Hosken DJ (2011). Male attractiveness, fertility and susceptibility to oxidative stress are influenced by inbreeding in *Drosophila simulans*. J Evol Biol.

[CR34] Oksanen J, Guillaume Blanchet F, Friendly M, Kindt R, Legendre P, McGlinn D, Minchin PR, O’Hara RB, Simpson GL, Solymos P, et al (2017) vegan: Community Ecology Package. R package version 2.4–2. https://CRAN.R-project.org/package=vegan

[CR35] Omer AD, Leigh TF, Granett J (1992). Insecticide resistance of greenhouse whitefly (Homoptera, Aleyrodidae) and fitness on plant hosts relative to the San-Joaquin valley (California) cotton agroecosystem. J Appl Entomol.

[CR36] Oppert B, Hammel R, Throne JE, Kramer KJ (2000). Fitness costs of resistance to *Bacillus thuringiensis* in the Indian meal moth, *Plodia interpunctella*. Entomol Exp Appl.

[CR37] Partridge L, Farquhar M (1983). Lifetime mating success of male fruit flies (*Drosophila melanogaster*) is related to their size. Anim Behav.

[CR38] Partridge L, Hoffmann A, Jones JS (1987). Male size and mating success in *Drosophila melanogaster* and *Drosophila pseudoobscura* under field conditions. Anim Behav.

[CR39] Pitnick S (1991). Male size influences mate fecundity and remating interval in *Drosophila melanogaster*. Anim Behav.

[CR40] Platt N, Kwiatkowska RM, Irving H, Diabate A, Dabire R, Wondji CS (2015). Target-site resistance mutations (kdr and RDL), but not metabolic resistance, negatively impact male mating competiveness in the malaria vector *Anopheles gambiae*. Heredity.

[CR41] R Core Team (2015) R: A language and environment for statistical computing. R Foundation for Statistical Computing, Vienna. URL http://www.R-project.org/

[CR42] Rivero A, Magaud A, Nicot A, Vezilier J (2011). Energetic cost of insecticide resistance in *Culex pipiens* mosquitoes. J Med Entomol.

[CR43] Rostant WG, Kay K, Wedell N, Hosken DJ (2015). Sexual conflict maintains variation at an insecticide resistance locus. BMC Biol.

[CR44] Rowland M (1991). Activity and mating competitiveness of Gamma-HCH dieldrin resistant and susceptible male and virgin female *Anopheles gambiae* and *An. stephensi* mosquitos, with assessment of an insecticide rotation strategy. Med Vet Entomol.

[CR45] Schmidt JM, Good RT, Appleton B, Sherrard J, Raymant GC, Bogwitz MR, Martin J, Daborn PJ, Batterham P, Goddard ME (2010). Copy number variation and transposable elements feature in recent, ongoing adaptation at the *Cyp6g1* locus. PLoS Genet.

[CR46] Smith DT, Hosken DJ, Rostant WG, Yeo M, Griffin RM, Bretman A, Price TAR, ffrench-Constant RH, Wedell N (2011). DDT resistance, epistasis and male fitness in flies. J Evol Biol.

[CR47] Spieth HT (1974). Courtship behavior in *Drosophila*. Annu Rev Entomol.

[CR48] Stewart C (2016). An approach to measure distance between compositional diet estimates containing essential zeros. J Appl Stat.

[CR49] Tang JD, Collins HL, Roush RT, Metz TD, Earle ED, Shelton AM (1999). Survival, weight gain, and oviposition of resistant and susceptible *Plutella xylostella* (Lepidoptera: Plutellidae) on broccoli expressing CrylAc toxin of *Bacillus thuringiensis*. J Econ Entomol.

[CR50] Taylor ML, Wedell N, Hosken DJ (2008). Sexual selection and female fitness in *Drosophila simulans*. Behav Ecol Sociobiol.

[CR51] Wang LM, Dankert H, Perona P, Anderson DJ (2008). A common genetic target for environmental and heritable influences on aggressiveness in *Drosophila*. Proc Natl Acad Sci USA.

[CR52] West LJ, Hankin RKS (2008). Exact tests for two-way contingency tables with structural zeros. J Stat Softw.

