# Supplementary figures and images for: Pleiotropic Effects of DDT Resistance on Male Size and Behaviour
Source: Behav Genet. 2017 May 2;47(4):449–58. doi: 10.1007/s10519-017-9850-6 (PMC5486851; doi:10.1007/s10519-017-9850-6)

a) Susceptible male courtship

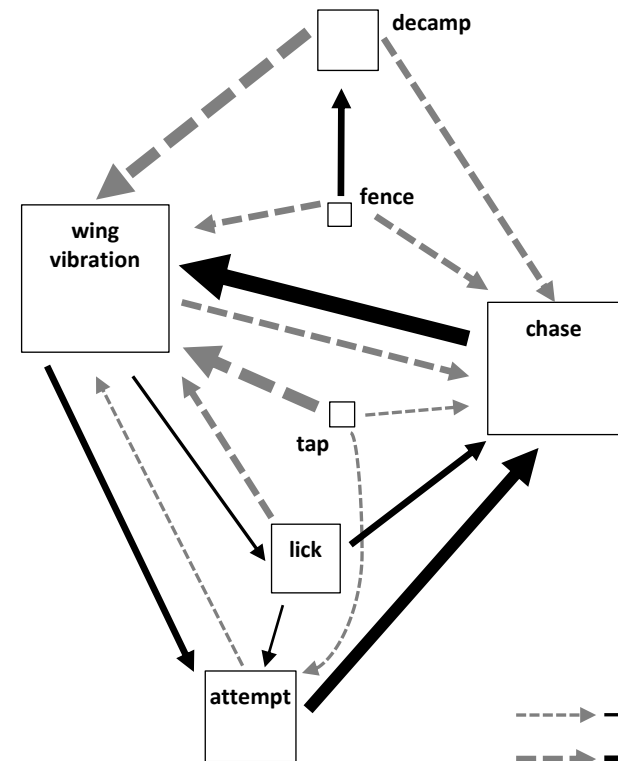

b) Resistant male courtship

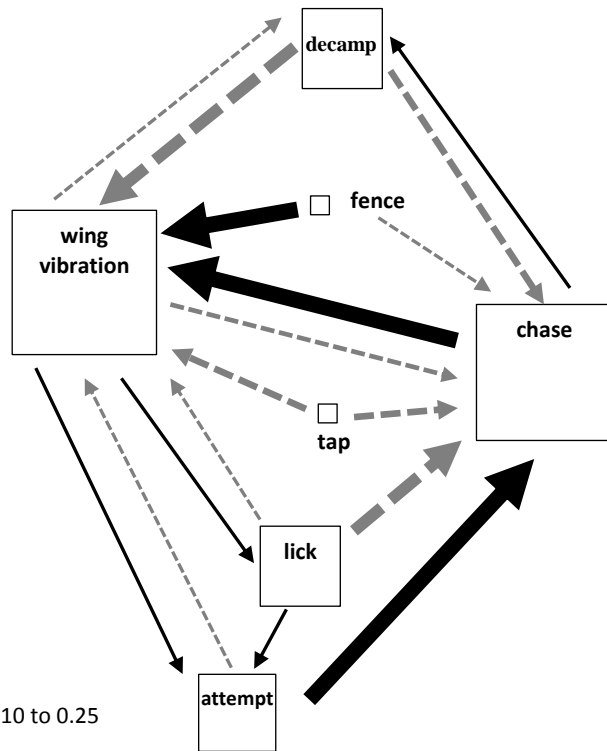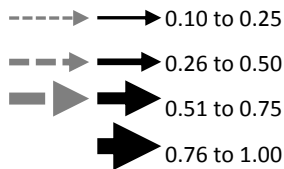

Supplement: Supplementary file 1 — Fig. S1 Kinematic diagram of behavioural transitions that occurred more than 10% of the time for (a) susceptible males and (b) resistant males during courtship. Arrow thickness indicates probability of occurrence. Solid, black arrows represent those transitions which occurred more frequently than expected by chance (p < 0.05) and grey dashed arrows show non-significant transitions (p < 0.05). Box size indicates frequency of behaviour. (PDF 182 KB) [file 10519_2017_9850_MOESM1_ESM.pdf]
