# Supplementary material for: Pleiotropic Effects of DDT Resistance on Male Size and Behaviour
Source: Behav Genet. 2017 May 2;47(4):449–58. doi: 10.1007/s10519-017-9850-6 (PMC5486851; doi:10.1007/s10519-017-9850-6)
Supplement: Supplementary file 2 — Supplementary material 2 (DOCX 12 KB) [file 10519_2017_9850_MOESM2_ESM.docx]

**Table S1.** Behaviours displayed by male *D. melanogaster* in courtship and aggression assays.

| **Designation** | **Behaviour** (as defined in *Ejima and Griffith 2007 and  †Chen et al. 2002) |
| --- | --- |
| Courtship:  Tapping * | Male touches female’s body with his foreleg |
| Fencing | Male and female hit each other’s forelegs |
| Chasing | Male follows female |
| Wing vibration * | Male extends and vibrates wing, producing courtship song |
| Genitalia licking * | Male extends proboscis and licks the female’s genitalia |
| Attempted copulation * | Male grasps female with forelegs and curls tip of his abdomen |
| Decamping | Male walks away from female, so no courtship is taking place |
| Aggression†: |  |
| Wing threat | Male quickly raises both wings to a 45° angle towards opponent |
| Lunging | One male rears up on hind legs and snaps down on the other |
| Holding | One male grasps the opponent with forelegs and tries to immobilize |
| Tussling | Both males tumble over each other, sometimes leaving food surface |
