# Supplementary material for: Pleiotropic Effects of DDT Resistance on Male Size and Behaviour
Source: Behav Genet. 2017 May 2;47(4):449–58. doi: 10.1007/s10519-017-9850-6 (PMC5486851; doi:10.1007/s10519-017-9850-6)
Supplement: Supplementary file 3 — Supplementary material 3 (DOCX 12 KB) [file 10519_2017_9850_MOESM3_ESM.docx]

**Table S2.** Overall behavioural transition matrix for resistant male courtship showing the count of each transition summed over 26 replicate trials. Transitions which occurred more frequently than by chance, as tested using a modified version of Fisher’s Exact test (see text) are indicated in bold. Structural zeros are indicated by dashes.

|  | **Following behaviour** | | | | | | | |
| --- | --- | --- | --- | --- | --- | --- | --- | --- |
| **Preceding behaviour** | attempt  copulation | chase | decamp | fence | lick | tap | wing vibration | **Row**  **Totals** |
| attempt  copulation | - | **60** | 7 | - | - | - | 11 | 78 |
| chase | 5 | - | **62** | **1** | 2 | 2 | **246** | 318 |
| decamp | 0 | 45 | - | 0 | - | 1 | 66 | 112 |
| fence | - | 1 | 0 | - | - | - | **5** | 6 |
| lick | **20** | 42 | 5 | - | - | - | 15 | 82 |
| tap | 0 | 3 | - | - | - | - | 3 | 6 |
| wing vibration | **79** | 162 | 38 | - | **80** | 2 | - | 361 |
| **Column**  **Totals** | 104 | 313 | 112 | 1 | 82 | 5 | 346 | 963 |
