# Supplementary material for: Pleiotropic Effects of DDT Resistance on Male Size and Behaviour
Source: Behav Genet. 2017 May 2;47(4):449–58. doi: 10.1007/s10519-017-9850-6 (PMC5486851; doi:10.1007/s10519-017-9850-6)
Supplement: Supplementary file 4 — Supplementary material 4 (DOCX 12 KB) [file 10519_2017_9850_MOESM4_ESM.docx]

**Table S3.** Overall behavioural transition matrix for susceptible male courtship showing the count of each transition summed over 26 replicate trials. Transitions which occurred more frequently than by chance, as tested using a modified version of Fisher’s Exact test (see main text) are indicated in bold. Structural zeros are indicated by dashes.

|  | **Following behaviour** | | | | | | | |
| --- | --- | --- | --- | --- | --- | --- | --- | --- |
| **Preceding behaviour** | attempt  copulation | chase | decamp | fence | lick | tap | wing vibration | **Row**  **Totals** |
| attempt  copulation | - | **57** | 4 | - | - | - | 16 | 77 |
| chase | 8 | - | 16 | 0 | 3 | 0 | **192** | 219 |
| decamp | 2 | 13 | - | **3** | - | **2** | 26 | 46 |
| fence | - | 2 | **2** | - | - | - | 3 | 7 |
| lick | **11** | **29** | 2 | - | - | - | 17 | 59 |
| tap | 1 | 2 | - | - | - | - | 5 | 8 |
| wing vibration | **81** | 110 | 22 | - | **56** | 3 | - | 272 |
| **Column**  **Totals** | 103 | 213 | 46 | 3 | 59 | 5 | 259 | 688 |
